# Analysis of Face Stability during Excavation of Double-O-Tube Shield Tunnel

**DOI:** 10.1155/2013/781968

**Published:** 2013-09-23

**Authors:** Yuyou Yang, Qinghong Zhou, Hongan Li, Xuegang Huang, Xiaoming Tu

**Affiliations:** ^1^School of Engineering and Technology, China University of Geosciences (Beijing), Beijing 100083, China; ^2^Haikou Forest Farm, Kunming, Yunnan 650114, China

## Abstract

This paper focuses on the face stability analysis of Double-O-Tube shield tunnel. This kind of analysis is significant to ensure the safety of workers and reduce the influence on the surrounding environment. The key point of the stability analysis is to determine the supporting pressure applied to the face by the shield. A collapse failure will occur when the supporting pressure is not sufficient to prevent the movement of the soil mass towards the tunnel. A three-dimensional collapse failure mechanism was presented in this paper. Based on the mechanism of a single circular shield tunnel, the mechanism of Double-O-Tube shield tunnel was established by using the fact that both of the mechanisms are symmetrical. Then by means of the kinematic theorem of limit analysis, the numerical results were obtained, and a design chart was provided. The finite difference software FLAC3D was applied to investigate the face failure mechanism of DOT shield tunnel established in this paper; the critical supporting pressures of the collapse failure mechanism in different strata (sand and silt) were calculated. Through comparative analysis, the theoretical values were very close to the numerical values. This shows that the face failure mechanism of DOT shield tunnel is reasonable, and it can be applied to the sand and silt strata.

## 1. Introduction

Compared with the single circular shield tunnel, the Double-O-Tube (DOT) shield tunnel has many advantages, for example, (i) optimizing, use of the underground space, (ii) saving construction cost and time, and (iii) reducing the impact on nearby structures. In 1989, Japan took the lead in adopting the DOT shield technology for a subway project in Hiroshima [1]. In the following ten years, more than ten DOT shield tunnels have been built in Japan, for example, Nagoya express railway tunnel; then, China first applied the DOT shield technology in the construction of Shanghai rail transit projects from 2002 to 2006 [2, 3]. In 2009, the Taoyuan International Airport Access Mass Rapid Transit system in Taipei, Taiwan, was constructed by the DOT shield machine [4]. With the rapid development of underground engineering in urban area, there will be more DOT shield tunnels in the future.

The stability analysis of a tunnel driven by a pressurized shield is a key issue in real shield tunneling projects. During the construction of a tunnel, the collapse failure at the cutter face will lead to the settlement of ground surface, which may cause damage to the surrounding buildings. Ensuring tunnel face safety requires the determination of the supporting pressure applied to the face by the shield. The DOT shield is an earth pressure balance type shield, in which the supporting pressure is applied by controlling the moving speed of screw. Investigation of the face stability of circular tunnels has been conducted by several authors in the literature. Leca and Dormieux proposed two mechanisms for the collapse failure; one contained a single conical block; the other one was composed of two conical blocks [5]. Soubra et al. proposed an optimized mechanism which was composed of five conical blocks that allow the 3D slip surfaces to develop more freely [6]. Then the mechanism by Mollon et al. was generated “point by point” instead of the simple use of existing standard geometric shapes such as cones or cylinders. All of these significant approaches are translational three-dimensional failure mechanisms and are based on the kinematical approach of limit analysis [7]. In addition, some scholars have studied this issue by experiments and numerical methods. Chambon and Corte carried out the centrifuge tests; the experimental results show that the collapse failure surface is similar to a chimney that does not necessarily outcrop at the ground surface [8]. Yang and Li proposed a three-dimensional collapse failure mechanism, and investigated the ground surface settlements and failure mechanism above large-diameter shield tunnels under different supporting pressures [9]. Yang et al. did some research about soil improvement techniques to guarantee the stability of excavation face [10, 11]. In order to observe the contour of failure surface, Takano et al. used X-ray computed tomography scanner; it was suggested that the contour of the failure surface can be simulated with two log-spirals in the vertical cross-sections and elliptical contour in the horizontal cross-sections. These experiments are beneficial to establish the more optimized analytical mechanisms [12]. Wei et al. used the finite element software MIDAS/GTS to simulate the construction process of the Double-O-Tube (DOT) shield tunnel and analyzed the building-soil-tunnel interaction [13].

This paper focuses on the face stability analysis of DOT tunnels. The biggest challenge of this kind of analysis is to establish a failure mechanism. It is necessary to note that the generation of the mechanism associating with the DOT tunnel can be developed from that of a single circular tunnel. This can be achieved if one realized that both the mechanisms are symmetrical. Detailed modeling progress of the DOT shield tunnels will be described later. The face stability analysis requires the determination of the supporting pressure that is applied to the tunnel face by the shield to ensure the stability of tunnel face and limit environmental impact. A three-dimensional collapse failure mechanism was presented, and the kinematical approach of the limit analysis theory was used to calculate the critical supporting pressures. A design chart was provided in the aim of computing the collapse pressures expediently. Then the finite difference software FLAC3D was applied to investigate the face failure mechanism of DOT shield tunnel established in this paper, and the results were presented.

## 2. Limit Analysis in Tunnel Face Stability

Limit analysis was first presented in terms of the theorems by Drucker et al. to estimate the critical height of slope; it also can be used to evaluate bounds on the limit load inducing or resisting failure in structures built of perfectly plastic materials [14, 15]. The aim of tunnel face stability analysis is to ensure that the supporting pressure is sufficient to pretend the soil mass collapse in front of the tunnel face. In fact, the key point of the analysis is to determine the lower bound pressures. Hence, the face stability analysis is a typical limit state problem. The kinematic theorem of limit analysis is known as the “upper bound theorem” because it can be stated as “the power of external loads applied to the structure is larger than the power that dissipated in the structure during its failure”. Generally it can be described in the following, mathematical form:

(1) ∫VD.(ε.ij)dV≥∫SvTividSv+∫StTividSt+∫VγividV.

The left side of (1) represents the rate of work dissipation during an incipient failure of a structure, and the right side includes the work rates of all external forces. *T*_*i*_ is the stress vector on boundaries *S*_*v*_ and *S*_*t*_. Vector *T*_*i*_ is unknown (limit load) on *S*_*v*_, and it is known on *S*_*t*_ (for instance, surcharge pressure). *v*_*i*_ is the velocity vector in the kinematically admissible mechanism, *γ*_*i*_ is specific weight vector, and *V* is the volume of the mechanism [16]. The collapse failure will occur if the supporting pressure is lower than the critical pressure. In this case, the supporting pressure is not a load, because it prevents the failure to appear. This can explain the puzzle: the “upper bound theorem” is applied to calculate the lower bound critical pressure.

In order to apply the limit analysis, some assumptions must be given: (i) the strain rates resulting from the velocity field must satisfy the flow rule; (ii) the failure must comply with the Mohr-Coulomb yield condition, which contains two material constants: the internal friction angle *φ* and the cohesion intercept *c*. According to the normality condition for an associated flow rule Coulomb material, for a kinematically admissible failure mechanism, the velocity discontinuity along a plastically deformed surface must make an angle *φ* with this velocity discontinuity surface [17]. 

In this paper, the external loads acting on the collapse failure mechanism contain: (i) the self-weight of the soil; (ii) the pressure *σ*_*t*_ acting on the tunnel face; (iii) the possible surcharge loading *σ*_*s*_ acting on the ground surface (if the mechanism outcrops). Then the kinematic theorem of limit analysis can be written in the following mathematical form:

(2) D=Wγ+Wσt+Wσs,

where *D* represents the rate of internal energy dissipation, *W*_*γ*_, *W*_*σ*_*t*__, and *W*_*σ*_*s*__ represent the rate of work of the soil self-weight, the rate of work of the applied pressure, and the rate of work of the possible surcharge, respectively.

The rate of work of the soil weight is calculated from a general expression as in

(3) Wγ=∭VviγidV,

where *v*_*i*_ and *γ*_*i*_ are the velocity vector and the unit weight vector, respectively, and *V* are the volume of the mechanism (below the ground surface).

The Rate of work of the pressure *σ*_*t*_ is calculated from a general expression as in

(4) Wσt=−σt∬SvidS=−σtvcos⁡βAT,

where *A*_*T*_ are the area of intersection of the tunnel face with the lower cone.

The rate of work of the possible uniform surcharge *σ*_*s*_ acting on the ground surface is calculated from a general expression as in

(5) Wσs=σs∬SvidS=σsvsinβAS,

where *A*_*S*_ are the possible area of the intersection of the mechanism with ground surface.

Considering that the mechanism is rigid, the only source of energy dissipation is derived from the plastic soil deformation that occurs along the velocity discontinuity surface. The rate of internal energy dissipation is calculated from a general expression as in

(6) D=∬Scvicos⁡φdS,

where S is the superficial area of the mechanism (below the ground surface). It should be mentioned here that the computation of energy dissipation could be made using an alternative convenient approach (for more details, see [17]).

By equating the total rate of external forces (3)–(5) to the total rate of internal energy dissipation (6), the pressure *σ*_*t*_ can be expressed as follows:

(7) σt=γDNγ+σsNs−cNc,

where *N*_*γ*_, *N*_*c*_, and *N*_*s*_ are nondimensional coefficients, representing, respectively, the effect of soil self-weight, cohesion, and surcharge loading.

## 3. The 3D Collapse Failure Mechanism

### 3.1. Geometrical Construction of the 3D Failure Mechanism

**Figure 1:**
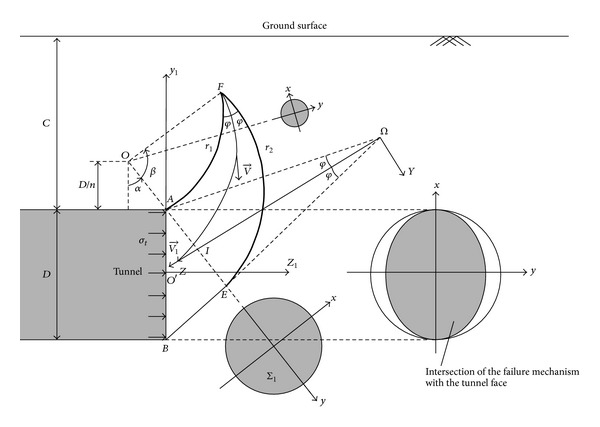
Cross-section of the collapse failure mechanism associating with a single circular tunnel in the (*y*, *z*) plane.

As mentioned previously, the generation of the mechanism associating with the DOT tunnel is based on that of a single circular tunnel. To better introduce the geometrical construction of the 3D failure mechanism of the DOT tunnel, it is necessary to introduce that of a single circular tunnel at first.  Figure 1 shows the cross-section of the mechanism in the vertical plane passing through the tunnel axis. The diameter of the tunnel is *D*, and *C* represents the cover depth. As shown in Figure 1, the mechanism is composed of a truncated conical block and a rotational curvilinear cone (the shape of the cone is like a “horn”), and the “horn” is located on the conical block; the contacting cross-section Σ_1_ is circular. The opening angle of the conical block is equal to 2*φ*. The rotational curvilinear cone can be described by two log spirals *r*_1_ and *r*_2_, which emerg from *A* and *E*, respectively, and share a common center *O*. The two log spirals intersect at point *F*. Their respective equations in a polar (*r*, *β*) coordinate system are as follows:

(8) r1=rA·exp⁡((α−β)·tanφ),r2=rE·exp⁡((β−α)·tanφ),

where *r*_*A*_ and *r*_*E*_ represent the distance between *O* and *A* and *E*, respectively. The central point *O* is located *D*/*n* above the tunnel as shown in Figure 1. Consider that

(9) rA=1ncos⁡α·D,rE=[1ncos⁡α+cos⁡(α+φ)cos⁡φ]·D.

The character of the “horn” contains: (i) each radial cross-section is circular with the diameter (*r*_2_ − *r*_1_); (ii) the apex angle of the horn equals 2*φ*; (iii) there is a vertical symmetry plane passing through the tunnel axis. The mechanism is kinematical admissibility, which requires along the entire sliding surface the velocity of the rotating soil to be inclined at angle *φ* to the surface; this assures that the dilatancy (volume increase) of the shearing soil is accommodated by the mechanism. This dilatancy is the direct consequence of the Mohr-Coulomb yield condition, and the normality flow rule. To satisfy this admissibility requirement the apex angle of the “horn” needs to be equal to 2*φ*. It is obvious that the shape of the mechanism depends on three variables: *φ*, *n*, and *α*.

**Figure 2:**
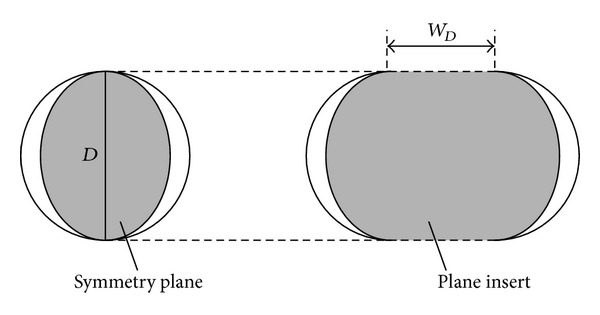
The evolutionary progress of the collapse failure mechanism from a single circular tunnel to DOT tunnel.

Then, based on the study of single-circular tunnel, the mechanism can be modified with plane inserts, to ensure transition to a plane mechanism with an increase in the width of the insert. The generating progress of the mechanism associating with the DOT tunnel is shown in Figure 2. Exciting the vertical symmetry plane allows the mechanism to extend along the width direction.

### 3.2. Velocity Field

At first, the velocity in the rotation section is introduced. The velocity direction is normal to the radial planes, and the magnitude is a function of radius *r*_*m*_ and angle *β* as shown in

(10) v→=ω→·rm,

where *ω* is the angular velocity about the axis passing through point *O*, and

(11) rm=r1+r22.

As shown in Figure 1, $\overset{\to }{v_{1}}$ is the velocity of the conical block. For the calculation of the rate of wok of these forces, $\overset{\to }{v_{1}}$ must be expressed in terms of the angular velocity *ω*. To solve this problem, one can use the fact that the contacting cross section Σ_1_ is circular. The velocity of point *I*, which is the centre of plane Σ_1_, is shown in

(12) vI→=ω→·rI,

where

(13) rI=rA+rE2=[1ncos⁡α+cos⁡(α+φ)2cos⁡φ]·D.

At the same time, considering the fact that $\overset{\to }{v_{I}}=\overset{\to }{v_{1}}$, then the expression of $\overset{\to }{v_{1}}$ can be obtained as follows:

(14) v1→=ω→·[1ncos⁡α+cos⁡⁡(α+φ)2cos⁡φ]·D.

## 4. Numerical Results

### 4.1. Limit Supporting Pressures

Based on the kinematical approach of limit analysis theory in tunnel face stability, the previously mentioned three non-dimensional coefficients (*N*_*γ*_, *N*_*c*_, and *N*_*s*_) can be obtained. As mentioned previously, for the same *φ*, the mechanism is different along with the change of the values of *n* and *α*. However, the change of *N*_*γ*_ is tiny when *n* > 3.5. The results are optimized when *α* = 30°. So, the numerical results presented in this paper are obtained for *n* = 3.5, *α* = 30°. 

**Figure 3:**
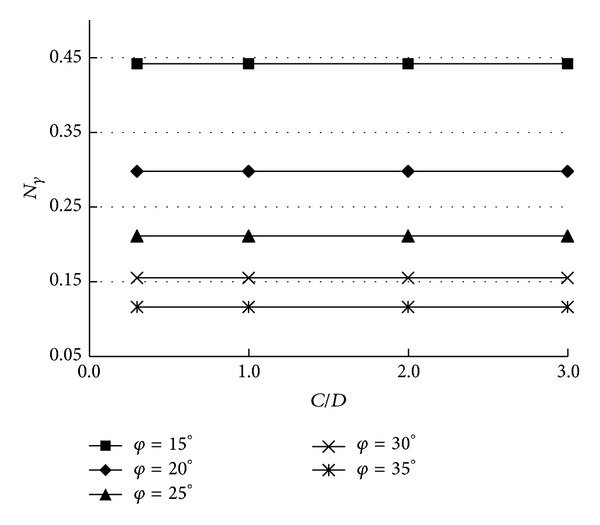
The values of *N*_*γ*_.

**Figure 4:**
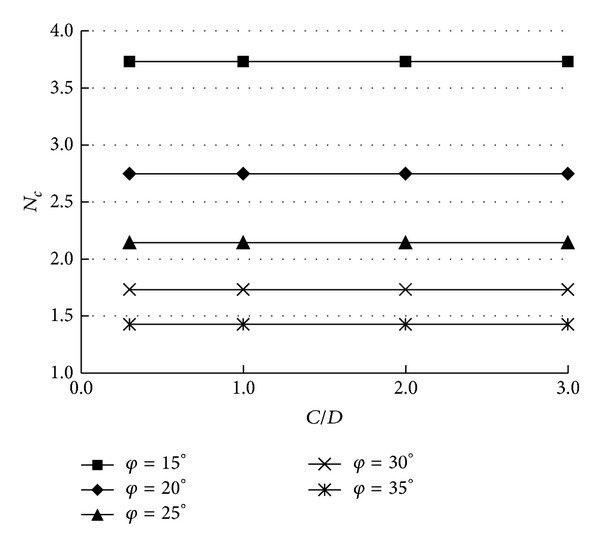
The values of *N*_*c*_.

The values of *N*_*γ*_ and *N*_*c*_ are provided in Figures 3 and 4, respectively.  Table 1 presents the collapse pressures for two drained clays: (i) *c* = 5 kPa and *φ* = 15° (soft clay); (ii) *c* = 12 kPa and *φ* = 30° (stiff clay). The presented critical collapse pressures are calculated for the diameter of the tunnel that is equal to 10 m, and *γ* = 18 kN/m^3^. It is necessary to note that when the overburden ratio is high enough (*C*/*D* > 0.5), the mechanism is always with nooutcrop. It is the reason why the coefficient *N*_*s*_ is equal to zero, and *N*_*γ*_ and *N*_*c*_ are constant with the increase of *C*/*D*. 

### 4.2. Design Chart

**Figure 5:**
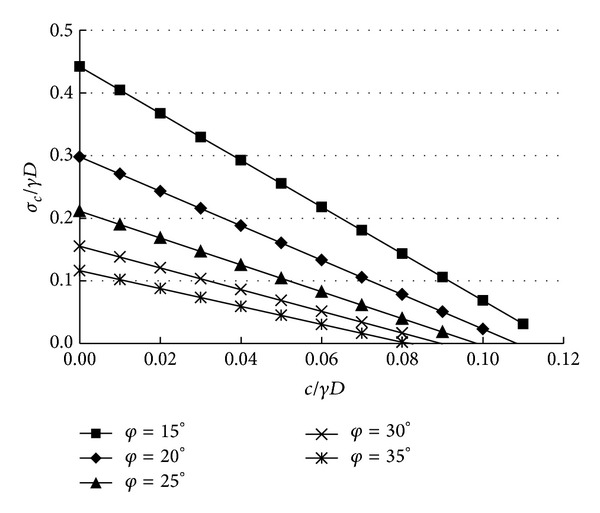
Design chart of critical pressure.

In order to make the numerical results associated with the proposed mechanism can be used expediently to compute the collapse pressures, a design chart is provided in Figure 3. The equation of each line in Figure 5 is presented in (15). It is obvious that the intercept on vertical coordinate represents the values of *N*_*γ*_, and the slope of the line represents the values of *N*_*c*_. Notice that the design chart also can be used to compute the required tunnel face supporting pressure when the safety factor *F*_*S*_ is given. The safety factor *F*_*S*_ is defined with respect to the two soil parameters *c* and tan *φ*. This may be achieved if one uses the chart with *c*_*d*_ and *φ*_*d*_ instead of *c* and *φ*, where *c*_*d*_ and *φ*_*d*_ are based on the following equations:

(15) σcγD=Nγ−cγD·Nc,

(16) cd=cFS,

(17) φd=arctan(tanφFS).

## 5. Numerical Analysis for Failure Mechanism of DOT Shield Tunnel Face

In order to verify the applicability of failure mechanism of DOT shield face established in this paper, the application of the failure mechanism used for sand and silt layers was analyzed, and the critical supporting pressures of collapse failure mechanism in different strata were calculated. And then the numerical results and theoretical results were comparatively analyzed to illustrate whether the failure mechanism is applicable. In the numerical calculation, the supporting pressure at the central of the tunnel face is taken to represent the magnitude of the face supporting pressure. In order to facilitate the description, the conception of supporting pressure ratio (SPR) is introduced to measure the magnitude of the face supporting pressure [18], namely,

(18) λ=σtσ0,

where *σ*_*t*_ is thesupporting pressure of tunnel face, *σ*_0_ is the earth pressure at rest of tunnel face.

### 5.1. Simulation of the Construction Mechanical Behavior of DOT Shield Tunnel

In this paper, the collapse failure of tunnel face and surface settlement caused by the improper supporting pressure during the shield tunnel construction are investigated to calculate the critical supporting pressure of tunnel face when the collapse failure occurs. Therefore, the finite difference software FLAC3D, which can reflect the large deformation of the rock mass, was applied to simulate the excavation process of DOT shield tunnel. The lagrangian method follows the assumption of a continuous medium; it allows the medium to large deformation and can reflect the geometric large deformation problems. 

During the shield tunnel construction, the soil excavation, shield driving, segment installation, and tail grouting are a continuous cycle. The influence of different supporting pressure on the excavated surface deformation, destructions and the surface subsidence is the focus of the analysis in this paper. Therefore, the excavation process is simulated using a simplified single-step excavation scheme, assuming that the tunnel is excavated a certain distance instantaneously and the supporting structure is installed. And then, the face supporting pressure is reduced gradually; the influence of the magnitude of the face supporting pressure on the surrounding soil deformation is investigated. Such a simplified modeling scheme has been successfully adopted in previous studies [19, 20]. When grouting at shield tail, cement slurry fills gaps and infiltrates into soil mass then gradually hardens. In this process, soil and cement slurry form a mixture, whose mechanical properties are affected by nature of soil, cement slurry material, and amount and pressure of grouting. In real analysis, it is very difficult to analyze quantitatively these factors. Taking into account the effects of the shield tail void, the degree of filling grout and the disturbance of soil around the tunnel on the ground deformations, the conception of equivalent circle zone is used to simulate the shield tail grouting, which is a layer of homogeneous and continuous elements representing the shield tail void between the outer surface of lining and the overexcavation surface [21].

### 5.2. Three-Dimensional Model and Parameters

**Figure 6:**
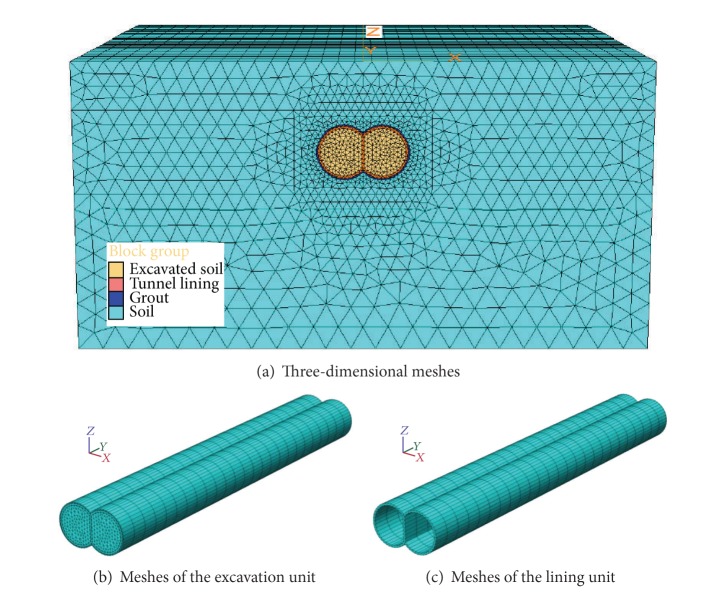
Meshes of the computing model.

According to the construction conditions of DOT shield tunnel at home, the tunnel excavation diameter is 6.5 m, depth is 7.5 m, the center distance is 4.6 m, segment is the C50 reinforced concrete material, and thickness is 0.3 m. In order to reduce the influence of the model size on the numerical results, the lateral length of the three-dimensional model is 80 m, the longitudinal length is 60 m, and the height is 35 m. The model has 28,652 nodes and 52,850 units. Three-dimensional model meshes, excavation unit, and lining unit are shown in Figure 6. The surface boundary in the present model is free, while the bottom boundary of the model is fully fixed; in four vertical boundaries, all horizontal displacements normal to the boundary are constrained to be zero, and the vertical displacement is free. The tunnel is constructed from the vertical start boundary at *y* = 0 m in the positive *y*-direction. An elastic perfectly plastic constitutive model based on the Mohr-Coulomb criterion is adopted to simulate deformation, strength, and failure behavior of the soil; the lining is modeled using continuous elements, and it is governed by a linear-elastic behavior. Physical and mechanical parameters of the materials are shown in Table 2.

### 5.3. Numerical Results Analysis

In this paper, two formations of sand and silt are considered; the influences of different magnitudes of face supporting pressure on the horizontal displacement and failure form of DOT shield tunnel face and surface deformation are investigated. In the numerical simulations, the continuity and circularity of shield construction are ignored, tunnel is excavated 25 m one time along the positive direction of the *y*-axis; at the same time, the supporting structure is timely installed and the shield tail is grouting. And then face supporting pressure is reduced gradually, until the collapsed failure of tunnel face occurs; the impact of magnitude of face supporting pressure on the surrounding soil deformation is investigated to obtain the critical supporting pressure of tunnel face.

#### 5.3.1. Analysis of the Failure Forms of the Tunnel Face

**Figure 7:**
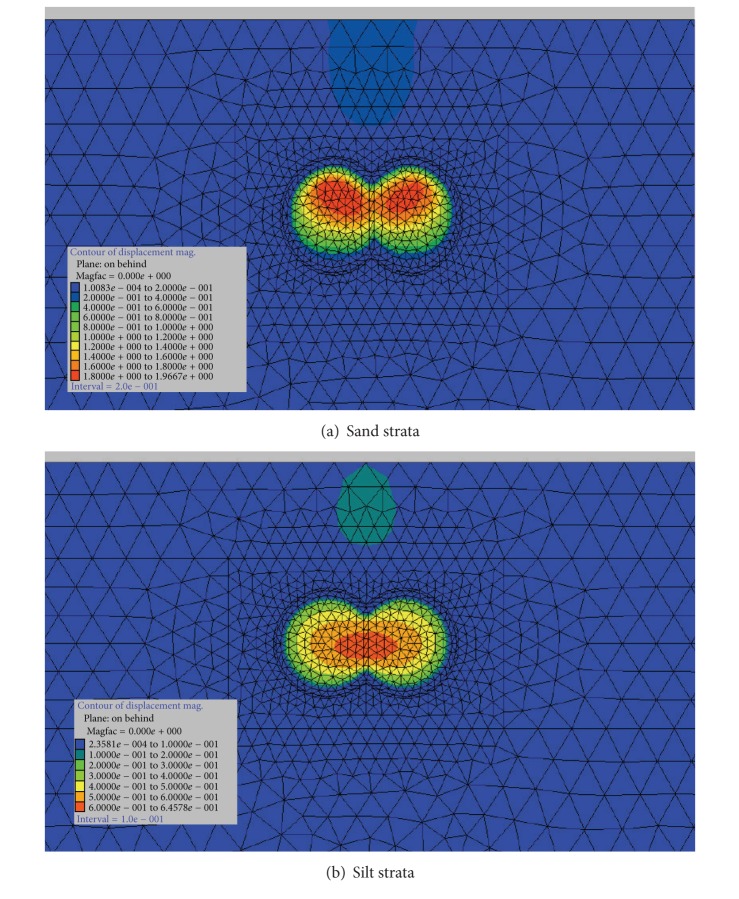
Cross-sectional failure form of the tunnel face.

**Figure 8:**
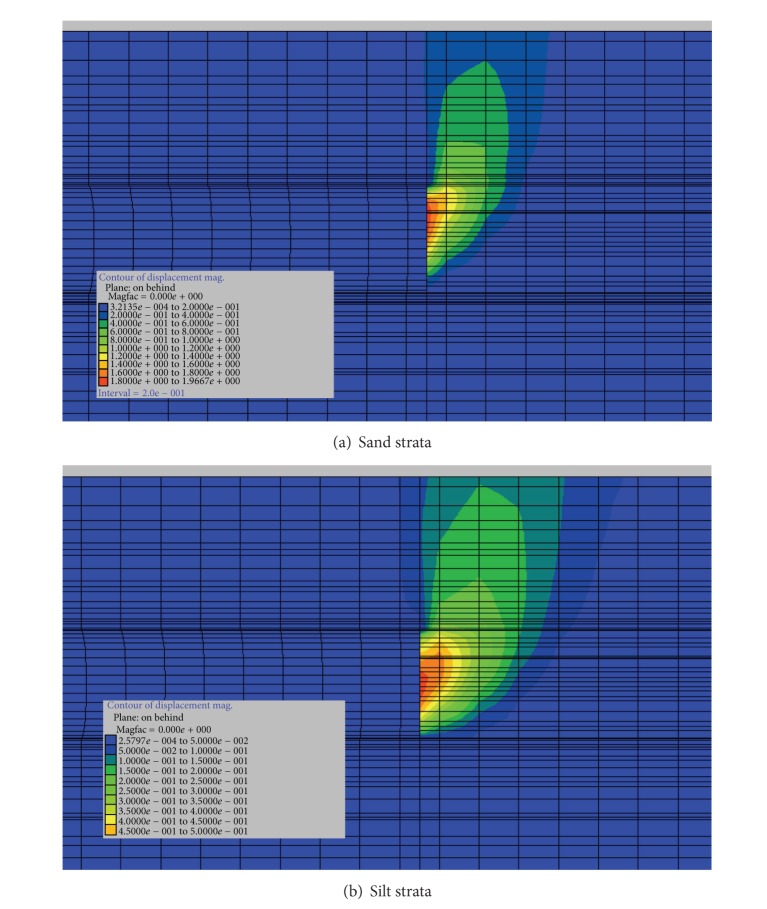
Longitudinal section failure form of the tunnel face.

Figures 7 and 8 show the failure forms of the tunnel face after the face collapse of DOT shield tunnel. From Figure 7, the collapse failure occurs at the top of tunnel face in the sand strata; however, the collapse failure occurs at the central of tunnel face in the silt strata. From Figure 8, it can be seen that, when the collapse failure tunnel face occur, the affected areas in sand strata are less than the affected areas in silt strata.

#### 5.3.2. Analysis of the Critical Supporting Pressure of the Tunnel Face

In shield tunneling, in order to reduce the soil disturbance ahead of the tunnel face, the supporting pressure applied in the tunnel face should be equal to the in situ stress at the tunnel face (i.e., the earth pressure at rest). When the face supporting pressure is less than the earth pressure at rest, the face deformation occurs towards the inside of the tunnel, in extreme cases, the face collapse failure occurs when the face supporting pressure is larger than the earth pressure at rest; the compressional deformation of soil in front of tunnel face occurs, and the ground surface appears uplift. In this numerical calculation, the earth pressure at rest at the center of the tunnel face was 100 kPa. The face supporting pressure was reduced gradually, and the maximum horizontal displacement of the tunnel face was calculated, until the maximum horizontal displacement of tunnel face suddenly increases sharply; therefore, the face supporting pressure at this time was considered as the critical supporting pressure causing the collapse failure of tunnel face.

**Figure 9:**
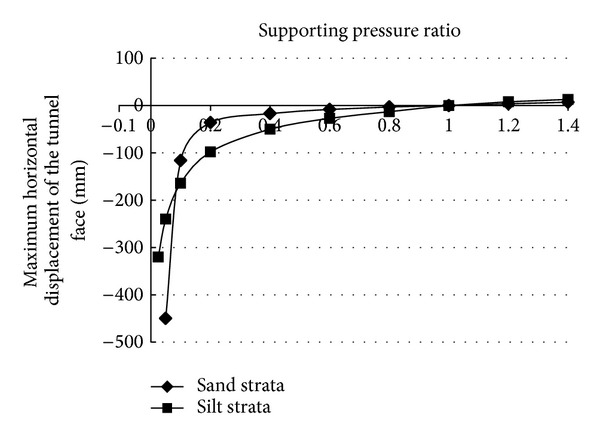
Relationship curve between the supporting pressure ratio and the maximum horizontal displacement of the tunnel face. Note: a negative value indicates the deformation towards inside the tunnel; a positive value indicates the compressional deformation in front of the tunnel face.

Figure 9 shows the relationship curve between the supporting pressure ratio and the maximum horizontal displacement of the tunnel face. When the face support pressure is less than the earth pressure at rest, with the decrease of the supporting pressure, the face deformation towards the inside of the tunnel gradually increases; when the face support pressure is greater than the earth pressure at rest, with the increase of the supporting pressure, the compressional deformation in front of the tunnel face gradually increases. In the sand strata, when the supporting pressure ratio is of 0.2–1.0, the growth rate of the maximum horizontal displacement of the tunnel face is almost consistent; when the supporting pressure ratio *λ* < 0.2, the maximum horizontal displacement of the tunnel face increases sharply. Therefore, in the sand strata, when the face collapse failure of DOT shield tunnel occurs, the critical supporting pressure ratio *λ* is considered to be 0.2; namely, the critical supporting pressure is 20 kPa. In the silt strata, when the supporting pressure ratio *λ* is 0.1–1.0, the growth rate of the maximum horizontal displacement of the tunnel face increases gradually; when the supporting pressure ratio *λ* < 0.1, the maximum horizontal displacement of the tunnel face increases sharply. Therefore, in the silt strata, when the face collapse failure of DOT shield tunnel occurs, the critical supporting pressure ratio *λ* is considered to be 0.1; namely, the critical supporting pressure is 10 kPa. When the face supporting pressure is greater than the critical supporting pressure, decreasing the face supporting pressure, the face deformation is small, and the face deformation in the silt strata is greater than that in the sand strata. When the face supporting pressure is less than the critical supporting pressure, in the sand strata, decreasing supporting pressure slightly, the face deformation increases rapidly, or the face collapse failure occurs; in the silt strata, decreasing supporting pressure slightly, the face deformation increases rapidly, but the growth rate of the horizontal displacement of the tunnel face is less than the growth rate of the horizontal displacement in the sand strata.

From Figure 9, it can be seen that the deformation and failure of the tunnel face caused by the change of the supporting pressure applied in the DOT shield tunnel face can be divided into three stages. The first stage: when the face supporting pressure is greater than the earth pressure at rest, the compressional deformation of soil in front of tunnel face occurs. The second phase: when the face supporting pressure is located between the earth pressure at rest and critical supporting pressure, the face deformation caused by decreasing the supporting pressure is small. The third stage; when the face supporting pressure is less than the critical supporting pressure, decreasing the face supporting pressure slightly will cause a significant deformation or the collapse failure of the DOT shield tunnel face.

It can be seen from Table 3, in the sand strata, that the theoretical result of the critical supporting pressure is slightly larger than the numerical result when the face collapse failure of the DOT shield tunnel occurs, but the both are close; in the silt strata, the theoretical result is very close to the numerical result. Therefore, the face collapse failure mechanism of the DOT shield tunnel established in this paper is reasonable, and it can be applied in the sand and silt strata.

#### 5.3.3. Analysis of the Ground Settlement after the Face Collapse Failure

**Figure 10:**
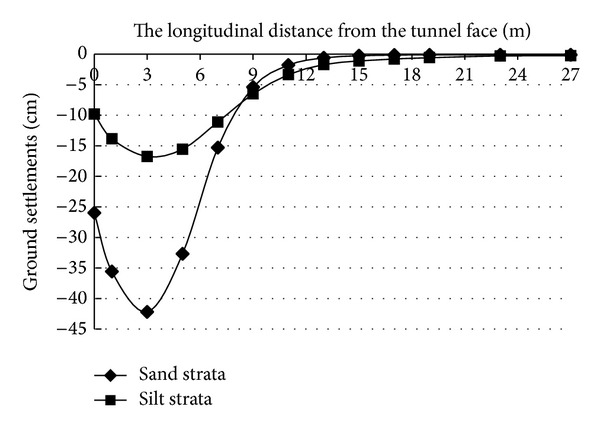
The longitudinal ground settlements ahead of tunnel face after collapse failure of the tunnel face.

In order to investigate the ground settlement after the destruction of the tunnel face, the longitudinal and transverse subsidence monitoring points were laid on the ground surface in front of tunnel face.  Figure 10 shows the longitudinal ground settlements after the face collapse failure occurs. The figure shows that there is a V-shaped settlement trough in sand strata after the face collapse failure of the DOT shield tunnel occurs. However, there is a U-shaped settlement trough in silt strata after the face collapse failure of the DOT shield tunnel occurs; the maximum settlement occurs in the front side about 3 m of tunnel face (approximately *D*/2; *D* is the diameter of the tunnel). The maximum ground settlement value in the sand strata is greater than that in the silt strata; however, the sphere of influence on the ground in the sand strata is smaller than in the silt strata.

**Figure 11:**
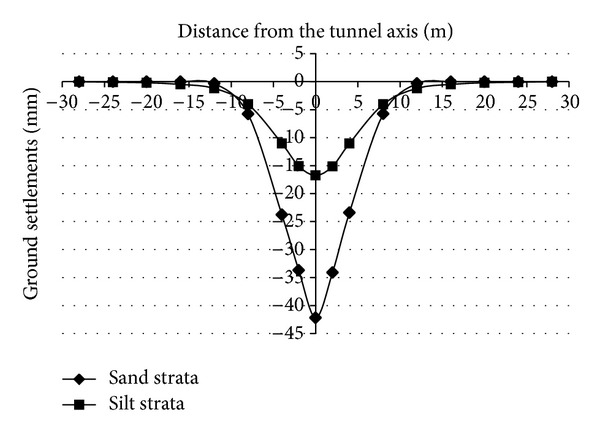
The transverse ground settlements ahead of tunnel face after collapse failure of the tunnel face.

In order to conduct a study on the transverse ground settlements, the transverse ground settlements in the front side about 3 m of tunnel face (the largest settlement position) were monitored.  Figure 11 shows the transverse ground settlements after the face collapse failure occurs. The figure shows that there is a V-shaped settlement trough in sand strata after the face collapse failure of the DOT shield tunnel occurs. However, there is a U-shaped settlement trough in silt strata after the face collapse failure of the DOT shield tunnel occurs, and the width of the settlement trough in the silt strata is larger than the width of the settlement trough in the sand strata. 

#### 5.3.4. Effects of Supporting Pressure on the Ground Settlements

**Figure 12:**
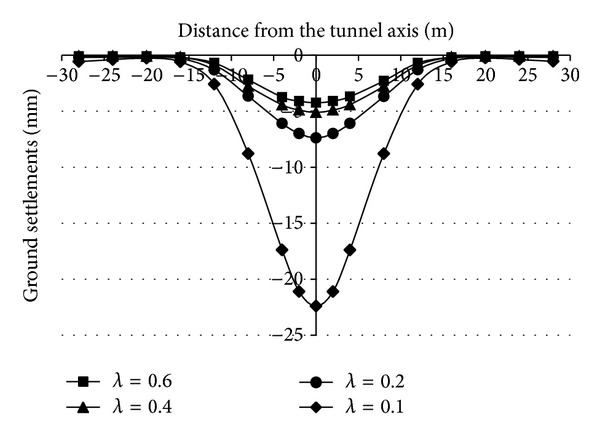
Relation curves between the supporting pressure and the horizontal ground settlements in the sand strata.

**Figure 13:**
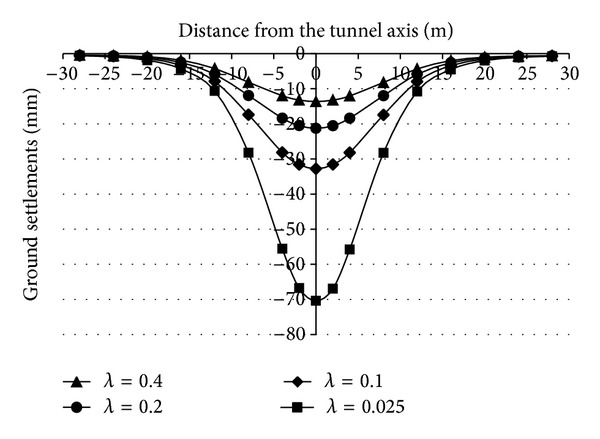
Relation curves between the supporting pressure and the horizontal ground settlements in the silt strata.

It is important to take the relation between the face supporting pressure and the ground settlements for the projects, especially the changes of ground settlements when the supporting pressure is smaller than the critical supporting pressure. Figures 12 and 13 are the relation curves between the supporting pressure ratio and the transverse ground settlements in the sand strata and silt strata. 

Figures 12 and 13 show that, when the supporting pressure is larger than the critical supporting pressure (*λ* > 0.2 or 0.1), the changes of ground settlements induced by decreasing the supporting pressure are not large and when the supporting pressure is less than the critical supporting pressure (*λ* < 0.2 or 0.1), decreasing the supporting pressure slightly makes the ground settlements become large sharply. 

## 6. Conclusions

A three-dimensional collapse failure mechanism associated with the DOT shield tunnel was presented in the aim to calculate the critical pressure. Exciting the rotational “horn” in the mechanism allows the slip surface to develop more freely than the mechanism composed of conical blocks. The kinematical approach of the limit analysis theory was used to calculate the critical pressures. A design chart in terms of the parameters *φ*, *c*/*γD*, and *σ*_*c*_/*γD* was provided, which can be used to determine the lower bound pressure applied to the tunnel face. 

The finite difference software FLAC3D was applied to investigate the face failure mechanism of DOT shield tunnel established in this paper; the critical supporting pressure of the collapse failure mechanism in different strata (sand and silt) were calculated. Analytical results show that the face failure mechanism of DOT shield tunnel is reasonable, and it can be applied to the sand and silt stratas.

## Figures and Tables

**Table 1:** Critical collapse pressures of given soil parameters.

	Soft clay (*c* = 5 kPa, *φ* = 15°)	Stiff clay (*c* = 12, *φ* = 30°)
*N*_*γ*_	0.442	0.155
*N*_*c*_	3.732	1.732
*N*_*s*_	0	0
*σ*_*c*_	60.878 kPa	7.175 kPa

**Table 2:** Physical and mechanical parameters of the materials.

Material type	Thickness (m)	Bulk density (kN/m^3^)	Cohesion (kN)	Friction angle (°)	Elastic modulus (MPa)	Poisson's ratio
Sand	—	18	0	25	25	0.34
Silt	—	18	10	15	15	0.33
C50 reinforced concrete lining	0.3	25	—	—	34500	0.17
Tail grouting	0.15	16.5	—	—	1.2	0.2

**Table 3:** Critical supporting pressure of the DOT shield tunnel.

Strata	Theoretical results (kPa)	Numerical results (kPa)
Sand	23	20
Silt	10.7	10
